# Circulating Transcriptional Profile Modulation in Response to Metabolic Unbalance Due to Long-Term Exercise in Equine Athletes: A Pilot Study

**DOI:** 10.3390/genes12121965

**Published:** 2021-12-09

**Authors:** Katia Cappelli, Samanta Mecocci, Stefano Capomaccio, Francesca Beccati, Andrea Rosario Palumbo, Alessia Tognoloni, Marco Pepe, Elisabetta Chiaradia

**Affiliations:** 1 Department of Veterinary Medicine, University of Perugia, 06126 Perugia, Italy; katia.cappelli@unipg.it (K.C.); samanta.mecocci@studenti.unipg.it (S.M.); francesca.beccati@unipg.it (F.B.); fotodarp@gmail.com (A.R.P.); alessia.tognoloni@studenti.unipg.it (A.T.); marco.pepe@unipg.it (M.P.); elisabetta.chiaradia@unipg.it (E.C.); 2 Sports Horse Research Center, University of Perugia, 06126 Perugia, Italy

**Keywords:** mi-RNA, physical exercise, gene expression, stress

## Abstract

Physical exercise has been associated with the modulation of micro RNAs (miRNAs), actively released in body fluids and recognized as accurate biomarkers. The aim of this study was to measure serum miRNA profiles in 18 horses taking part in endurance competitions, which represents a good model to test metabolic responses to moderate intensity prolonged efforts. Serum levels of miRNAs of eight horses that were eliminated due to metabolic unbalance (Non Performer-NP) were compared to those of 10 horses that finished an endurance competition in excellent metabolic condition (Performer-P). Circulating miRNA (ci-miRNA) profiles in serum were analyzed through sequencing, and differential gene expression analysis was assessed comparing NP versus P groups. Target and pathway analysis revealed the up regulation of a set of miRNAs (of mir-211 mir-451, mir-106b, mir-15b, mir-101-1, mir-18a, mir-20a) involved in the modulation of myogenesis, cardiac and skeletal muscle remodeling, angiogenesis, ventricular contractility, and in the regulation of gene expression. Our preliminary data open new scenarios in the definition of metabolic adaptations to the establishment of efficient training programs and the validation of athletes’ elimination from competitions.

## 1. Introduction

While regular physical activity is known to be beneficial for health, prolonged or high-intensity exercise can cause stress due to duration and inability of humans and horses to adapt physiologically to these conditions [1,2,3,4,5].

Competitive sports are known to be demanding and stressful for both human and animal athletes. In particular, endurance is one of the most challenging among equestrian disciplines; endurance horses are susceptible to metabolic imbalance due to dehydration, acid balance and electrolyte abnormalities, substrate depletion and heat accumulation, which can result in life-threatening conditions [6]. Indeed, equine endurance competitions, which have recently gained popularity worldwide, are governed by the rules established by National and International Equestrian Federations (FEI), which implement strict regulations to safeguard and ensure the welfare of animals [7].

Endurance horses are subjected to specific training that induces the physiological adaptations required for carrying out prolonged moderate intensity exercise on different types of ground surfaces and under different weather conditions, by modulating their energy metabolism towards aerobic conditions [8]. However, when horses are poorly trained, or high speeds are required, anaerobic pathways may be recruited. Endurance athletes also develop hypervolemia, which, when coupled with splanchnic vasoconstriction physiologically triggered by stress hormones, enables maintaining of good central blood pressure and satisfactory perfusion of the main organs. Prolonged physical exercise can also induce changes in coagulation systems, immune modulation, and vascular integrity, and is known to have negative effects on health and welfare [9]. Indeed, the homeostatic disruptions that occur during long competitions have been suggested as the main causes of certain pathological conditions such as myopathies, colic, laminitis, diaphragmatic flutter, cardiac arrhythmias and massive rhabdomyolysis [7]. Therefore, it is essential to find early biomarkers that can promptly identify subjects at risk and prevent dysmetabolism.

Evidence has been found that alterations in circulating miRNA (ci-miRNA) expression are induced by physical exercise and endurance [10,11,12].

Among the RNAs, micro-RNAs (miRNAs) are highly conserved regulatory molecules that play active roles in cell differentiation, proliferation and metabolism. MiRNAs drive post-transcriptional down-regulation and bind to mRNAs, with a one-to-many (and vice versa) relationship with their targets, in that a gene can be regulated by different miRNAs, and the same miRNA can regulate different genes [13].

MiRNAs can be released into the bloodstream within apoptotic bodies, extracellular vesicles (i.e., exosomes), high/low-density lipoproteins (HDL and LDL) or as active protein complexes (RNA-binding proteins) [14,15]. Stable ci-miRNAs can be found in plasma and/or in serum, many of which are tissue-specific and signatures of certain physiological and/or pathological conditions [12]. Physical exercise has recently been associated with the modulation of small noncoding RNAs in the bloodstream depending on type and duration of the physical activity [16]. Therefore, ci-miRNAs have been proposed as biomarkers for evaluating human athletic performance and were recently used in our previous study on endurance riding [17]. 

The aim of this research is to acquire a deeper molecular knowledge of responses to prolonged moderate-intensity exercise in endurance horses and to identify ci-miRNAs related to nonphysiological responses. To this aim, the ci-miRNA transcriptional profiles of horses that successfully finished an endurance race were compared using high-throughput sequencing and those with severe metabolic disorders after a competition were eliminated. Our hypothesis is that differentially modulated ci-miRNA in horses after a race elimination could provide clues to a reduced adaptation to exercise stress leading to low performance syndromes and diseases. We therefore tried to obtain a transcriptional picture of the multiorgan response and gain a better understanding of the molecular basis of this phenomenon.

## 2. Materials and Methods

### 2.1. Ethics Statement

The study protocol was reviewed and approved by institutional Ethics Committee of University of Perugia (license No. 2019-32). All procedures were performed in accordance with the approved guidelines. Owner informed consent and the approval of the Ground Jury President and the Veterinary Commission President of the event were obtained before initiation of study procedures with the animals.

In order to correctly report research on live animals, the manuscript was prepared following the ARRIVE guidelines (https://arriveguidelines.org/, accessed on 8 December 2021).

### 2.2. Sample Collection

For this study, eighteen Arabian horses, thirteen females, four geldings and one male, aged from 6 to 13 years old (median 9 years old) engaged in 80–160 km national and international competitions held in the same season of 2018 in Italy were recruited. During competition at least every 40 km there is a compulsory veterinary inspection (vet gate) to determine if the horse is fit to continue as indicated by FEI Endurance Rules (available at: https://inside.fei.org/fei/disc/endurance/rules, accessed on 8 December 2021) including irregular gait (i.e., lameness) or metabolic reasons, such as failure to recovery maximum heart rate (i.e., 64 beats per minutes) cardiac arrhythmia, clinical signs of metabolic instability, excessive fatigue, heat stroke, colic, myopathy, severe dehydration or excessively high temperature by evaluation of heart, gut sound, mucous membrane colour, capillary refill time and muscle tone.

The horses enrolled were divided into two groups based on veterinary gates inspection results: Performer (P) was composed of 10 subjects that successfully completed the competition at free speed and Non Performer (NP) constituted the remaining eight subjects that were eliminated at different stages of the competition (Table S1).

Peripheral blood was collected from the jugular vein using a vacutainer with and without anticoagulant at the end of race or at the intermediate veterinary examination that resulted in elimination of the horse from the competition within 30′. The serum samples were obtained by centrifugation at 400× *g* in a bench-top centrifuge for 15 min, immediately after collection and then stored at −80 °C until biochemical tests and miRNA isolation.

### 2.3. Blood Count and Biochemical Analysis 

A complete blood count with leukocyte differential assessment was performed using a laser haematology analyser (Sysmex XT-2000iV; Sysmex, Kobe, Japan). The analysis included white blood cells (WBCs), red blood cells (RBCs), mean corpuscular volume (MCV), haemoglobin (Hb), platelets (PLTs) and mean platelet volume (MPV), haematocrit (HCT), mean corpuscular haemoglobin (MCH), mean corpuscular haemoglobin concentration (MCHC) and red blood cell distribution width (RDW). Selected biochemical parameters were also analyzed with an Hitachi 904 automated biochemistry analyzer (Boehringer Mannheim, Baden-Wurttemberg, Germany) including urea, creatinine, total bilirubin (Tbil), aspartate aminotransferase (AST), γ-glutamyltransferase (GGT), creatine kinase (CK), lactate dehydrogenase (LDH), total proteins (TPs) and albumin (Alb). For blood count and biochemical analysis, Student’s *t*-test was applied to identify differences between continuous data between group NP and group P.

### 2.4. RNA Extraction and Library Preparation

Total RNA extraction was carried out using the commercial miRNeasy Serum/Plasma Advanced kit (Qiagen, Venlo, The Netherlands) following manufacturer’s instructions. For better evaluation, the miRNA differences between the two groups, spike-in sequences were added to the lysis buffer at the beginning of the RNA extraction procedure (2.5 µL of Spike-in solution per 600 µL of serum for each subject) using the QIAseq miRNA Library QC Spike-in kit (Qiagen, Venlo, The Netherlands), which provides 52 spike-in phosphorylated at 5′, an essential feature for the library preparation [18]. Adding these plant origin sequences allowed us to obtain quality control for the sequencing process and to normalize the results in terms of the number of sequenced fragments for each miRNA. The RNA quantity and quality was evaluated with NanoDrop 2000 (Thermo Fisher Scientific, Waltham, MA, USA) spectrophotometer and microfluidic electrophoresis respectively (Bioanalyzer 2100 Agilent Technologies, Santa Clara, CA, USA). The TruSeq Small RNA Library Illumina (Illumina Inc., 5200 Illumina Way, San Diego, CA, USA) kit was used for library construction following the manufacturer’s instructions, and fragments were sequenced on a NextSeq500 Illumina (Illumina Inc., 5200 Illumina Way, San Diego, CA, USA) instrument with 75bp single-end chemistry. 

### 2.5. Bioinformatic Analysis

**Figure 1:**
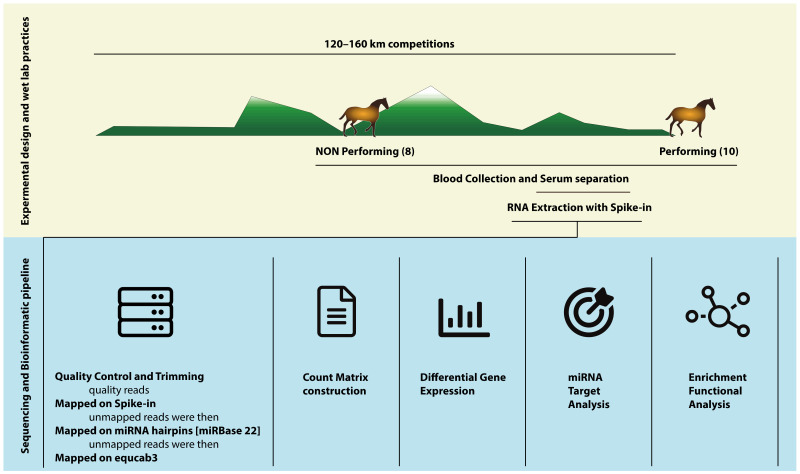
Experimental design (cream background) and bioinformatic pipeline (cadet blue background).

Raw reads in fastq format were quality checked with FastQC (https://www.bioinformatics.babraham.ac.uk/projects/fastqc/, accessed on 8 December 2021) and trimmed with Trim Galore 0.5.0 software for the removal of low-quality sequences and adapters (https://www.bioinformatics.babraham.ac.uk/projects/trim_galore/, accessed on 8 December 2021). The Bowtie2 [19] aligner of the Tuxedo suite was used to align cleaned reads adopting a three-step alignment strategy: (i) on the spike-in set used in the experiment; (ii) on the mirbase 22 hairpin database (Release 22.1) (http://www.mirbase.org, accessed on 8 December 2021) for the unmapped reads on spike-in and (iii) on the horse reference genome (equcab3) [20], for the unmapped on miRNA database (Figure 1). After the alignment procedure, the dataset was normalized for spike-ins data through the RUVSeq [21] R package. Briefly, RUVSeq uses spike-in reads to extrapolate correction coefficients for the samples that are integrated, among other confounding effects (i.e., sex), into the differential gene expression (DGE) evaluation analysis model implemented in edgeR [22]. A PCA plot from expression data is available in Figure S1. Expression levels were measured as count per million (CPM) and parameters set for DGE comparing the NP to the P group with an absolute log2-fold change (logFC) > 1.5 and an adjusted *p*-value for multiple testing correction applying the Benjamini-Hochberg method (FDR) < 0.05.

#### Target Genes and Enrichment Analysis

Human or murine orthologue of all the differentially expressed miRNAs, according to the DGE analysis from edgeR, were retrieved using miRbase (http://www.mirbase.org, accessed on 8 December 2021) software and used to identify putative genes (predicted and/or validated) targeted by these miRNAs. The most frequently sequenced human or murine miRNA form IDs (3p or 5p) were selected as input in the miRWalk 3.0 webtool (http://mirwalk.umm.uni-heidelberg.de, accessed on 8 December 2021). A unique list of predicted and validated targets was identified for each miRNA, specifying also the miRNA site of action with respect to the targeted mRNA: 3′UTR, 5′UTR or CDS (coding region). Only the genes targeted by five or more miRNAs were selected for the downstream analyses.

The Cytoscape 3.7.1 suite [23] was used to build a Protein-Protein Interaction Network (PPI) using the IMEx database, which contains nonredundant information derived from the major public protein databases. The clusterMaker 2.0 a Cytoscape application [24] with the “gLay” option was used to highlight different clusters within the network based on the number and type of connections between the nodes. Clusters with a number of interactions greater than 30 were inspected for Gene Ontology (GO) enrichment analysis carried out for the related biological process through the BiNGO application [25]. Results were filtered with a corrected FDR < 0.05 (Benjamini Hockberg correction). A summary of the experimental design and the bioinformatic pipeline is depicted in Figure 1.

## 3. Results

### 3.1. Hematology and Clinical Chemistry Analyses

The hematology and clinical chemistry assays evidenced values of hematocrit (*p* < 0.01), hemoglobin (*p* < 0.01), total protein, albumin (*p* < 0.01), urea (*p* < 0.05) and creatinine (*p* < 0.01) significantly higher in the Non Performer (NP) group compared to that of Performer (P) group whereas classical exercise biochemical markers such as creatine phosphokinase (CPK), aspartate-transaminase (AST) and lactic-dehydrogenase (LDH) values were not significantly different between the two groups, even after normalizing for the kilometers runs (Table S2).

### 3.2. Sequencing Statistics

The sequencing depth average was ~18,380,000 reads; of these, the 77.72% passed the trimming step. Two samples (P2 and P3) showed an unusually higher percentage (81% and 61% respectively) of discarded reads due to poor quality and/or length. Only the reads mapped exactly one time on miRBase 22 or EquCab3 annotated as sequences referable to miRNA were used for downstream analyses. Sequencing results and alignment rates are summarized in (Table S3).

### 3.3. Differential Expression Analysis of miRNA

After the statistical analysis with edgeR, starting from a cleaned dataset of 288 miRNAs, a total of seven differentially expressed (FDR < 0.05) were found. All of these were up-regulated (logFC ≥ +1.5) in the NP group compared to P group (Table 1).

### 3.4. miRNA-Target Evaluation and Pathway Analysis

Targets of each miRNA, for all the binding sites (3′-UTR, 5′-UTR and CDS of the target) were individualised from mirWalk analysis (Table 2). All the target lists were merged in order to produce a unique series of 16 genes that were selected according to the number of miRNAs they targeted, setting the threshold of at least five (Table 3).

**Figure 2:**
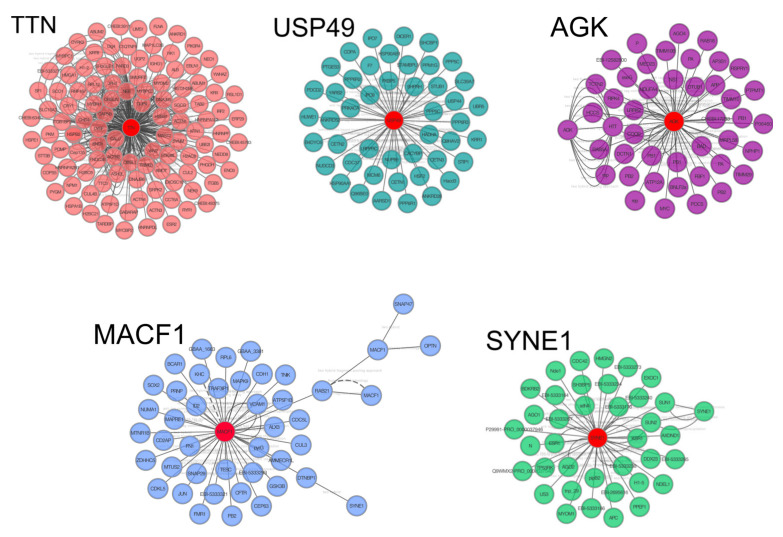
Cluster of proteins derived from clusterMaker 2.0 cytoscape application. Central nodes (red) indicate most relevant proteins. Clusters in boxes refer to networks between mouse proteins.

These genes were used to create a PPI network to identify clusters of proteins retrieved from the IMEx database. The total network contained 481 nodes and 657 edges; the major protein clusters, i.e., those with more than 30 interactions, are reported in Figure 2, where proteins are indicated of central nodes that correspond to target genes which have the highest number of interactions with other proteins correlated to similar biological functions. Gene Ontology (GO) enrichment analysis with BiNGO was performed on these major clusters (Table S4). The most enriched GO terms for the TTN cluster related to cardiac function and the apoptotic process such as “muscle structure development”, “muscle organ development”, “cytoskeleton organization”, “muscle contraction”, “cardiac myofibril assembly”, “regulation of apoptotic process” and “regulation of programmed cell death”. Regarding AGK cluster central node “mitochondrial transport”, “lactate biosynthetic process” and “negative regulation of glucocorticoid secretion” were among the main enriched terms. “Response to stimulus”, “response to stress”, “regulation of immune system process” together with “cell cycle”, “cell migration” and “cytoskeleton organization” were the main enriched GO terms for the cluster with MACF1 central node, while USP49 seemed to be related to protein folding. Terms related to translation regulation, such as “miRNA mediated inhibition of translation”, “regulation of translation, ncRNA-mediated” and “negative regulation of translation, ncRNA-mediated”, were among the main enriched biological processes found for the SYNE1 central nodes cluster. Complete data from GO analysis for all the PPI clusters are reported in Table S4.

## 4. Discussion

The molecular mechanisms underlying successful adaptation to stress associated with prolonged or high-intensity exercise remain poorly understood. In this study, the miRNA expression profiles of equine athletes competing in national and international endurance races were analysed. The differences observed in the number of circulating miRNAs between the nonperforming (NP group, eliminated by the competition) and performing equine athletes (P group, those who successfully finished the competition) were investigated.

Comparing the hematological and biochemical profiles of the two experimental groups, NP horses showed signs of severe dehydration, as they had higher values of hematocrit, hemoglobin, creatinine, total plasma proteins, albumins, and increased protein catabolism indicated by higher serum urea concentration. High levels of muscle enzyme markers (including for example creatine phosphokinase (CPK), aspartate-transaminase (AST) and lactic-dehydrogenase (LDH)) were found, as expected, in both groups’ serum (Table S2). However, no significant differences were observed among P and NP horses in enzymes. This confirmed the low correlation between these serum enzymes levels and the poor performance of equine athletes as suggested by other authors [26]. This evidence indicates that deeper knowledge is needed about molecular events modulating the response to physical stress that can also lead to metabolic alterations and low performance. miRNAs are ideal molecules for this purpose due to their properties and their fast response to physiological stress [12,16]. The results show that besides skeletal muscle tissues (SMTs), other tissues may be involved in an equine athlete’s adaptation to exercise-induced stress, since physiological stress induced by exercise triggers multiorgan responses in skeletal muscle, vasculature, heart and lung [27]. Garciarena et al. suggested that endurance training can improve cardiac function by “transforming” pathological cardiac hypertrophy into a physiological state [28] during which miRNAs have been described as key players [29]. However, it is important to note that NP horses were eliminated from the competition due to metabolic disorders including long recovery times.

As a whole, the results seem to indicate that the NP horses failed to adapt to exercise-induced stress. We observed, indeed, an increase in mir-101 plasma levels in these horses, which is in contrast to what was observed in trained horses [30]. Mirna-101 plays an important role in cardiac hypertrophy [31], and can be decreased by endurance training [32].

Interestingly, our results also showed high levels of some mir-17-92 cluster members, namely mir-18a, mir-20a and mir-106, which have been linked to myocardial ischemia/reperfusion injuries [31].

Increases in mir-20 and mir-106 levels have been also related to stress [33]. They are both involved in glucose metabolism regulation, while mir-106 has been linked to oxidative stress [33]. This has been proposed as a potential biomarker for recovery after exercise, as it was down-regulated following exhaustive endurance exercise and restored to normal levels within 2 h after the competition [34,35]. Moreover, over-expressed mir-106b has been associated with skeletal muscle mitochondrial dysfunction and insulin resistance, which are affected by exercise [34]. The high levels of mir-106b and mir-20a in our NP athletes also suggests endothelial function modulation failure [36] and the promotion of angiogenesis, that usually occurs during endurance training. Indeed, mir-106 exhibits anti-angiogenic properties, whereas mir-20a, also involved in angiogenesis, was found to quantitatively correlate with peak exercise capacity, cardiorespiratory fitness [37] and endothelial repair capacity [38]. However, increased expression of miR-20a was only observed at rest after sustained training and in response to hypertension-induced heart failure [39].

Mir-15b which was found to be increased in NP horses, is known to be an anti-angiogenic miRNA [40,41]. These miRNAs suppress vascular endothelial growth factor (VEGF) [40,41], basic fibroblast growth factor (bFGF) VEGF receptor 2 and FGF receptor 1 expression) [42]. The mir-15 family modulates cardiac hypertrophy, and its members are up-regulated during myocardial disorders [43]. Mir-451 is one of the most abundant miRNAs found in red blood cells [44,45] and was increased in NP horses. It was found to be increased in low responders to resistance exercise training [46] and has been correlated with coronary artery disease [47] in the occurrence of ischemic stroke.

Most of the modulated miRNAs in NP horses identified protein-coding genes as the targets involved in cardiac and skeletal muscle cytoskeleton, which is the main coordinator of muscle contraction [48]. More specifically, some proteins such as TTN (titin), SYNE1 (nesprin), and MACF1 (Microtubule Actin Cross-linking Factor 1), which are involved in nuclear and cytosolic cytoskeleton organization, were highlighted as being crucial cluster nodes by the PPI network analysis (Figure 2). Titin is involved in cardiac and skeletal muscle remodeling and modulates the elastic properties of the sarcomere as well as contractile muscle properties [49]. Furthermore, titin regulates myocardial passive stiffness and myocardial and ventricular function, mediates nuclear signaling and modulates muscle response to mechanical stress [50]. Moreover, the expression of various titin isoforms has been associated with sarcomere length, which correlates with muscle fascicle length and may enhance running performance.

Other cytoskeleton alterations are suggested by MACF, which is known to be a stress-induced regulator of cardiomyocyte microtubule distribution [48] essential for ventricular adaptation to hemodynamic overload [51], and to cardiac response to exercise. Muscle contraction could also be affected by changes in Ca^2+^ influx in our NP horses since *CACNA1B* (Voltage-dependent N-type calcium channel subunit alpha-1B), the gene target of some modulated miRNAs, was repressed and previous studies reported that many of the genes encoding ion channels are differentially methylated in horses and humans following exercise [52]. Moreover AGK (acylglycerol kinase) protein, a central cluster node evidenced by the PPI network analysis (Figure 2), is involved in the metabolism of mitochondrial phospholipids and in the stability of SLC25A4 (ADP/ATP translocase 1). Interesting, *SLC25A4* knockout mice exhibit phenotypes of hypertrophic cardiomyopathy, exercise intolerance, and lacticacidemia [53].

USP49, another PPI network central node, is involved in splicing alterations as it is essential for the cotranscriptional splicing [54]. Moreover, one of the target genes, *TDRD12*, is a gene expression enhancer via the production of secondary piRNAs. Since these small RNAs are key molecules in the transposable elements activation/expression [55], it is intriguing to think that one of the possible outcomes of this down regulation could be a genome plasticity response induced by exercise [3,56].

Overall, analysis of differentially expressed miRNAs and their target genes in NP horses identified genes were associated with the modulation of the various steps of gene expression essential for adaptation processes, namely “response to stimulus”, “regulation of translation”, “regulation of apoptotic process” and “muscle structure development”, underlying cardiac and skeletal muscle plasticity that occurs during prolonged physical activity and training [57].

## 5. Conclusions

For the NP horses, the results suggest a specific ci-miRNA profile pointing towards molecular mechanisms and metabolic pathways underlying the inability of tissues/organs to adapt to stress induced by prolonged physical exercise and training. Modulation of myogenesis, cardiac and skeletal muscle remodeling, angiogenesis, ventricular contractility and the regulation of gene expression appear to be the most involved processes.

It has to be said, though, that intrinsic limitations such as sample size and heterogeneity of recruited subjects due to the unpredictability of on-field collection, make this study a preliminary investigation. Validation with alternative techniques and a larger cohort of subjects, and possibly time course sampling, will be valuable in this intriguing research field.

## Figures and Tables

**Table 1:** Differentially expressed miRNAs (NP vs. P).

miRNA	logFC	logCPM	FDR
eca-mir-211	4.9223101	0.1280119	0.0015486
eca-mir-15b	4.6239495	3.7887133	0.0027720
eca-mir-451	2.1155572	9.8706133	0.0092029
eca-mir-18a	5.4076681	1.2325327	0.0092029
eca-mir-20a	3.2246991	2.7407456	0.0175767
eca-mir-106b	2.4277831	4.8328315	0.0213777
eca-mir-101-1	2.0112158	7.7859690	0.0213777

**Table 2:** Target gene consistencies of each miRNA detailed with respect to the binding site.

miRNA ID	Target 3′-UTR	Target 5′-UTR	Target CDS	Total Target
mir-101-3p	315	43	322	680
mir-106b-5p	1442	263	1435	3140
mir-15b-5p	1102	285	1623	3010
mir-18a-5p	1550	356	2242	4148
mir-20a-5p	1167	183	1038	2388
mir-211-5p	2161	844	2626	5631
mir-451-5p	171	44	409	624

**Table 3:** Target genes selected on the miRNA consensus analysis (recognized by at least five differentially expressed miRNAs).

Target Genes	miRNAs	# of miRNA
*TTN*	mir-101; mir-106b; mir-15b; mir-18a; mir-20a; mir-211	6
*MACF1*	mir-101; mir-106b; mir-15b; mir-18a; mir-20a	5
*SYNE1*	mir-101; mir-15b; mir-18a; mir-20a; mir-211	5
*TDRD12*	mir-106b; mir-15b; mir-18a; mir-20a; mir-211	5
*USP49*	mir-101; mir-106b; mir-15b; mir-18a; mir-211	5
*ACTR8*	mir-101; mir-106b; mir-15b; mir-18a; mir-211	5
*AGK*	mir-101; mir-106b; mir-15b; mir-20a; mir-211	5
*CACNA1B*	mir-106b; mir-15b; mir-18a; mir-20a; mir-211	5
*COL6A5*	mir-101; mir-106b; mir-15b; mir-18a; mir-211	5
*FAM227A*	mir-106b; mir-15b; mir-18a; mir-20a; mir-211	5
*HECW2*	mir-106b; mir-15b; mir-18a; mir-20a; mir-211	5
*PGM2L1*	mir-106b; mir-15b; mir-18a; mir-20a; mir-211	5
*PKD1L2*	mir-101; mir-106b; mir-18a; mir-20a; mir-211	5
*RIMBP2*	mir-106b; mir-15b; mir-18a; mir-20a; mir-211	5
*VPS13A*	mir-101; mir-15b; mir-18a; mir-20a; mir-211	5
*ZBTB37*	mir-101; mir-106b; mir-18a; mir-20a; mir-211	5

## Data Availability

Raw sequence data are available in SRA. BioProject ID collecting all samples is the following: PRJNA726388.

## Supplementary Materials

The following materials are available online at https://www.mdpi.com/article/10.3390/genes12121965/s1. Figure S1: Representation of the samples after the Principal Component analysis on miRNA features. Table S1: Details of the distance travelled, average speed and details of the veterinary inspection reported in the vet card at the time of completion (group P) or elimination (group NP). Table S2: Hematology and clinical chemistry values differences between groups P and NP. Table S3: Statistics of sequencing and mapping. Table S4: Multiple tables describing enrichment analysisfnot.

## References

[1] Dhabhar F.S. (2014). Effects of stress on immune function: The good, the bad, and the beautiful. Immunol. Res..

[2] Morton J.P., Kayani A.C., McArdle A., Drust B. (2009). The Exercise-Induced stress response of skeletal muscle, with specific emphasis on humans. Sports Med..

[3] Cappelli K., Mecocci S., Gioiosa S., Giontella A., Silvestrelli M., Cherchi R., Valentini A., Chillemi G., Capomaccio S. (2020). Gallop racing shifts mature mRNA towards introns: Does exercise-induced stress enhance genome plasticity?. Genes.

[4] Cappelli K., Amadori M., Mecocci S., Miglio A., Antognoni M.T., Razzuoli E. (2020). Immune response in young thoroughbred racehorses under training. Animals.

[5] Cappelli K., Felicetti M., Capomaccio S., Nocelli C., Silvestrelli M., Verini-Supplizi A. (2013). Effect of training status on immune defence related gene expression in Thoroughbred: Are genes ready for the sprint?. Vet. J..

[6] Noakes T.D. (2000). Physiological models to understand exercise fatigue and the adaptations that predict or enhance athletic performance. Scand. J. Med. Sci. Sports.

[7] Nagy A., Dyson S.J., Murray J.K. (2012). A veterinary review of endurance riding as an international competitive sport. Vet. J..

[8] Amory H., Votion D.M., Fraipont A., Goachet A.G., Robert C., Farnir F., Van Erck E. (2010). Altered systolic left ventricular function in horses completing a long distance endurance race. Equine Vet. J..

[9] Scoppetta F., Tartaglia M., Renzone G., Avellini L., Gaiti A., Scaloni A., Chiaradia E. (2012). Plasma protein changes in horse after prolonged physical exercise: A proteomic study. J. Proteom..

[10] Mooren F.C., Viereck J., Krüger K., Thum T. (2014). Circulating micrornas as potential biomarkers of aerobic exercise capacity. Am. J. Physiol. Heart Circ. Physiol..

[11] Polakovičová M., Musil P., Laczo E., Hamar D., Kyselovič J. (2016). Circulating MicroRNAs as potential biomarkers of exercise response. Int. J. Mol. Sci..

[12] Xu T., Liu Q., Yao J., Dai Y., Wang H., Xiao J. (2015). Circulating microRNAs in response to exercise. Scand. J. Med. Sci. Sports.

[13] Lombardi G., Perego S., Sansoni V., Banfi G. (2016). Circulating miRNA as fine regulators of the physiological responses to physical activity: Pre-analytical warnings for a novel class of biomarkers. Clin. Biochem..

[14] Turchinovich A., Weiz L., Langheinz A., Burwinkel B. (2011). Characterization of extracellular circulating microRNA. Nucleic Acids Res..

[15] Valadi H., Ekström K., Bossios A., Sjöstrand M., Lee J.J., Lötvall J.O. (2007). Exosome-mediated transfer of mRNAs and microRNAs is a novel mechanism of genetic exchange between cells. Nat. Cell Biol..

[16] Makarova J.A., Maltseva D.V., Galatenko V.V., Abbasi A., Maximenko D.G., Grigoriev A.I., Tonevitsky A.G., Northoff H. (2014). Exercise immunology meets MiRNAs. Exerc. Immunol. Meets MiRNAs.

[17] Cappelli K., Capomaccio S., Viglino A., Silvestrelli M., Beccati F., Moscati L., Chiaradia E. (2018). Circulating miRNAs as putative biomarkers of exercise adaptation in endurance horses. Front. Physiol..

[18] Head S.R., Kiyomi Komori H., LaMere S.A., Whisenant T., Van Nieuwerburgh F., Salomon D.R., Ordoukhanian P. (2014). Library construction for next-generation sequencing: Overviews and challenges. Biotechniques.

[19] Langmead B., Salzberg S.L. (2012). Fast gapped-read alignment with Bowtie 2. Nat. Methods.

[20] Kalbfleisch T.S., Rice E.S., DePriest M.S., Walenz B.P., Hestand M.S., Vermeesch J.R., O’Connell B.L., Fiddes I.T., Vershinina A.O., Saremi N.F. (2018). Improved reference genome for the domestic horse increases assembly contiguity and composition. Commun. Biol..

[21] Risso D., Ngai J., Speed T.P., Dudoit S. (2014). Normalization of RNA-seq data using factor analysis of control genes or samples. Nat. Biotechnol..

[22] Robinson M.D., McCarthy D.J., Smyth G.K. (2009). edgeR: A Bioconductor package for differential expression analysis of digital gene expression data. Bioinformatics.

[23] Shannon P., Markiel A., Ozier O., Baliga N.S., Wang J.T., Ramage D., Amin N., Schwikowski B., Ideker T. (2003). Cytoscape: A software Environment for integrated models of biomolecular interaction networks. Genome Res..

[24] Morris J.H., Apeltsin L., Newman A.M., Baumbach J., Wittkop T., Su G., Bader G.D., Ferrin T.E. (2011). ClusterMaker: A multi-algorithm clustering plugin for Cytoscape. BMC Bioinform..

[25] Maere S., Heymans K., Kuiper M. (2005). BiNGO: A Cytoscape plugin to assess overrepresentation of Gene Ontology categories in Biological Networks. Bioinformatics.

[26] Fielding C.L., Magdesian K.G., Rhodes D.M., Meier C.A., Higgins J.C. (2009). Clinical and biochemical abnormalities in endurance horses eliminated from competition for medical complications and requiring emergency medical treatment: 30 cases (2005–2006): Retrospective study. J. Vet. Emerg. Crit. Care.

[27] Vega R.B., Konhilas J.P., Kelly D.P., Leinwand L.A. (2017). Molecular Mechanisms Underlying Cardiac Adaptation to Exercise. Cell Metab..

[28] Garciarena C.D., Pinilla O.A., Nolly M.B., Laguens R.P., Escudero E.M., Cingolani H.E., Ennis I.L. (2009). Endurance training in the spontaneously hypertensive rat conversion of pathological into physiological cardiac hypertrophy. Hypertension.

[29] Feng H.J., Ouyang W., Liu J.H., Sun Y.G., Hu R., Huang L.H., Xian J.L., Jing C.F., Zhou M.J. (2014). Global microRNA profiles and signaling pathways in the development of cardiac hypertrophy. Braz. J. Med. Biol. Res..

[30] Faraldi M., Gomarasca M., Sansoni V., Perego S., Banfi G., Lombardi G. (2019). Normalization strategies differently affect circulating miRNA profile associated with the training status. Sci. Rep..

[31] Das A., Samidurai A., Salloum F.N. (2018). Deciphering Non-coding RNAs in Cardiovascular Health and Disease. Front. Cardiovasc. Med..

[32] Keller P., Vollaard N.B.J., Gustafsson T., Gallagher I.J., Sundberg C.J., Rankinen T., Britton S.L., Bouchard C., Koch L.G., Timmons J.A. (2011). A transcriptional map of the impact of endurance exercise training on skeletal muscle phenotype. J. Appl. Physiol..

[33] Solich J., Kuśmider M., Faron-Górecka A., Pabian P., Kolasa M., Zemła B., Dziedzicka-Wasylewska M. (2020). Serum Level of miR-1 and miR-155 as Potential Biomarkers of Stress-Resilience of NET-KO and SWR/J Mice. Cells.

[34] Håkansson K.E.J., Sollie O., Simons K.H., Quax P.H.A., Jensen J., Nossent A.Y. (2018). Circulating Small Non-coding RNAs as Biomarkers for Recovery After Exhaustive or Repetitive Exercise. Front. Physiol..

[35] Nielsen S., Åkerström T., Rinnov A., Yfanti C., Scheele C., Pedersen B.K., Laye M.J. (2014). The miRNA plasma signature in response to acute aerobic exercise and endurance training. PLoS ONE.

[36] Brown M.D., Hudlicka O. (2003). Modulation of physiological angiogenesis in skeletal muscle by mechanical forces: Involvement of VEGF and metalloproteinases. Angiogenesis.

[37] Baggish A.L., Hale A., Weiner R.B., Lewis G.D., Systrom D., Wang F., Wang T.J., Chan S.Y. (2011). Dynamic regulation of circulating microRNA during acute exhaustive exercise and sustained aerobic exercise training. J. Physiol..

[38] Wang D., Wang Y., Ma J., Wang W., Sun B., Zheng T., Wei M., Sun Y. (2017). MicroRNA-20a participates in the aerobic exercise-based prevention of coronary artery disease by targeting PTEN. Biomed. Pharmacother..

[39] Dickinson B.A., Semus H.M., Montgomery R.L., Stack C., Latimer P.A., Lewton S.M., Lynch J.M., Hullinger T.G., Seto A.G., Van Rooij E. (2013). Plasma microRNAs serve as biomarkers of therapeutic efficacy and disease progression in hypertension-induced heart failure. Eur. J. Heart Fail..

[40] Hua Z., Lv Q., Ye W., Wong C.K.A., Cai G., Gu D., Ji Y., Zhao C., Wang J., Yang B.B. (2006). Mirna-directed regulation of VEGF and other angiogenic under hypoxia. PLoS ONE.

[41] Triozzi P.L., Achberger S., Aldrich W., Singh A.D., Grane R., Borden E.C. (2012). The association of blood angioregulatory microRNA levels with circulating endothelial cells and angiogenic proteins in patients receiving dacarbazine and interferon. J. Transl. Med..

[42] Caporali A., Emanueli C. (2011). MicroRNA-503 and the Extended MicroRNA-16 Family in Angiogenesis. Trends Cardiovasc. Med..

[43] Tijsen A.J., Van der Made I., Van den Hoogenhof M.M., Wijnen W.J., Van Deel E.D., de Groot N.E., Alekseev S., Fluiter K., Schroen B., Goumans M.-J. (2014). The microRNA-15 family inhibits the TGFβ-pathway in the heart. Cardiovasc. Res..

[44] Kirschner M.B., Edelman J.J.B., Kao S.C.H., Vallely M.P., Van Zandwijk N., Reid G. (2013). The impact of hemolysis on cell-free microRNA biomarkers. Front. Genet..

[45] Doss J.F., Corcoran D.L., Jima D.D., Telen M.J., Dave S.S., Chi J.T. (2015). A comprehensive joint analysis of the long and short RNA transcriptomes of human erythrocytes. BMC Genom..

[46] Davidsen P.K., Gallagher I.J., Hartman J.W., Tarnopolsky M.A., Dela F., Helge J.W., Timmons J.A., Phillips S.M. (2011). High responders to resistance exercise training demonstrate differential regulation of skeletal muscle microRNA expression. J. Appl. Physiol..

[47] Ren J., Zhang J., Xu N., Han G., Geng Q., Song J., Li S., Zhao J., Chen H. (2013). Signature of circulating MicroRNAs As potential biomarkers in vulnerable coronary artery disease. PLoS ONE.

[48] Henderson C.A., Gomez C.G., Novak S.M., Mi-Mi L., Gregorio C.C. (2017). Overview of the muscle cytoskeleton. Compr. Physiol..

[49] Anderson B.R., Granzier H.L. (2012). Titin-based tension in the cardiac sarcomere: Molecular origin and physiological adaptations. Prog. Biophys. Mol. Biol..

[50] Lewinter M.M., Granzier H.L. (2014). Cardiac titin and heart disease. J. Cardiovasc. Pharmacol..

[51] Fassett J.T., Xu X., Kwak D., Wang H., Liu X., Hu X., Bache R.J., Chen Y. (2013). Microtubule Actin Cross-Linking Factor 1 Regulates Cardiomyocyte Microtubule Distribution and Adaptation to Hemodynamic Overload. PLoS ONE.

[52] Allen D.G., Lamb G.D., Westerblad H. (2008). Impaired calcium release during fatigue. J. Appl. Physiol..

[53] Chu B., Hong Z., Zheng X. (2021). Acylglycerol Kinase-Targeted Therapies in Oncology. Front. Cell Dev. Biol..

[54] Zhang Z., Jones A., Joo H.Y., Zhou D., Cao Y., Chen S., Erdjument-Bromage H., Renfrow M., He H., Tempst P. (2013). USP49 deubiquitinates histone H2B and regulates cotranscriptional pre-mRNA splicing. Genes Dev..

[55] Todd C.D., Deniz Ö., Taylor D., Branco M.R. (2019). Functional evaluation of transposable elements as enhancers in mouse embryonic and trophoblast stem cells. eLife.

[56] Capomaccio S., Verini-Supplizi A., Galla G., Vitulo N., Barcaccia G., Felicetti M., Silvestrelli M., Cappelli K. (2010). Transcription of LINE-derived sequences in exercise-induced stress in horses. Anim. Genet..

[57] Choi S., Liu X., Li P., Akimoto T., Lee S.Y., Zhang M., Yan Z. (2005). Transcriptional profiling in mouse skeletal muscle following a single bout of voluntary running: Evidence of increased cell proliferation. J. Appl. Physiol..

