# Supplementary material for: Circulating Transcriptional Profile Modulation in Response to Metabolic Unbalance Due to Long-Term Exercise in Equine Athletes: A Pilot Study
Source: Genes (Basel). 2021 Dec 9;12(12):1965. doi: 10.3390/genes12121965 (PMC8701225; doi:10.3390/genes12121965)
Supplement: Supplementary file 1 [file genes-12-01965-s001.zip › supplementary/FigureS1.pdf]

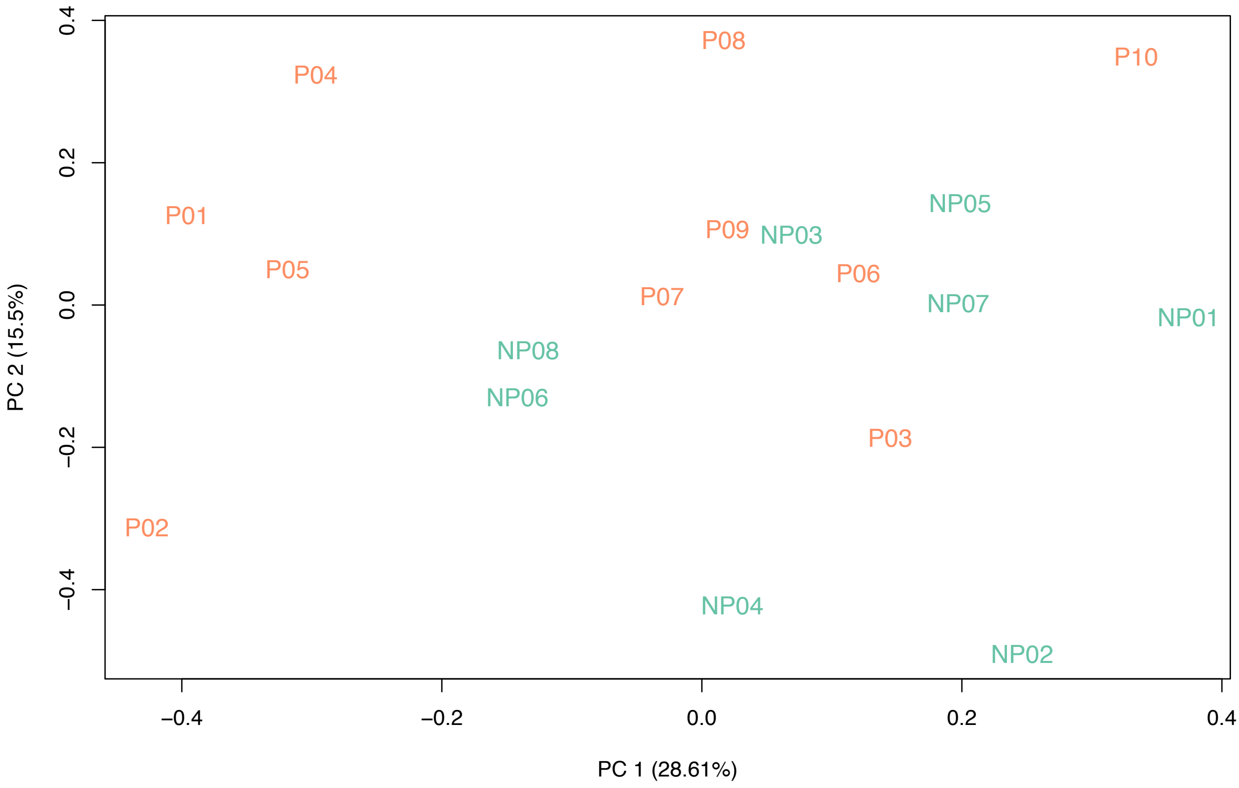

Figure S1. Principal Component Analysis based on miRNA expression values after spike-in correction of the two libraries: P (Performer) and NP (Non-Performer). The separation between the two groups was almost evident.
