# Supplementary material for: Circulating Transcriptional Profile Modulation in Response to Metabolic Unbalance Due to Long-Term Exercise in Equine Athletes: A Pilot Study
Source: Genes (Basel). 2021 Dec 9;12(12):1965. doi: 10.3390/genes12121965 (PMC8701225; doi:10.3390/genes12121965)
Supplement: Supplementary file 1 [file genes-12-01965-s001.zip › supplementary/Table_S1.pdf]

genes

MDPI

Horse	Distance travelled (km); class	Average speed (km/h)	Heart rate (bpm)	Cardiac Recovery index*	Respiratory	Mucous membrane colour	Capillary refill time (sec)	Skin test (sec)	Gut sounds	Lesions	Muscle tone	Gait
P1	94; CEI*	14,677	52	4	OK	B	2	1	A	A	A	A
P2	94; CEI*	14,588	54	2	OK	B	1	1	A	A	A	A
P3	84; CEN B	15,797	45	3	OK	A	1	1	A	A	A	B
P4	81; CEN B	16,003	54	2	OK	B	1	1	A	A	A	A
P5	81; CEN B	17,123	56	0	OK	B	1	2	A	A	A	A
P6	81; CEN B	14,143	62	0	OK	A	1	1	A	A	A	A
P7	91; CEI*	19,983	55	5	OK	B	2	2	A	A	A	A
P8	91; CEI*	18,343	50	2	OK	B	2	2	B	A	A	A
P9	81; CEN B	15,053	56	0	OK	B	1	2	A	A	A	B
P10	91; CEI*	16,139	54	2	OK	A	2	1	B	A	A	A
NP1	84; CEI***	17,433	69	na	OK	A	1	1	A	A	A	A
NP2	140; CEI***	13,106	75	na	OK	C	3	2	B	A	B	B
NP3	114; CEI***	14,531	57	7	OK	A	1	1	B	A	A	A
NP4	68; CEI***	13,701	52	8	OK	B	2	1	B	A	A	A
NP5	94; CEI***	14,646	67	na	OK	C	3	1	B	A	A	A
NP6	114; CEI***	14,094	75	na	OK	C	3	2	C	A	A	A
NP7	114; CEI**	16,607	76	na	OK	B	2	2	B	A	A	A
NP8	76; CEN B	16,625	60	8	OK	B	1	2	B	A	A	A

CEI = Concours de Raid d'Endurance International; * = 80-119 km; ** = 120-139 km; ***160 km. (Endurance Rules 2018).
CEN B = National Endurance Competition of 80-99 km.
Bpm = beats per minutes; Cardiac recovery index: difference between the first heart rate assessed after the entry in the vet gate and the second heart rate assessed after the evaluation of the gait in a straight line of 40 m trotting back and forth; na = not applicable as the horse was eliminated for high heart rate (metabolic) and cardiac recovery index is not reported in the vet card in these cases.
Respiratory evaluation consists in OK (normal) or abnormal.
Mucous membrane colour: A = normal; B = moderately hyperemic; C = severe congested
Gut sound: A = normal; B = decreased; C = absent. Lesions represents skin lesions: A = normal.
Muscle tone: A = normal; B = mildly increased; C = myopathy
Gait: A = sound; B = mildly irregular but fit to compete; C = irregular gait

Table S1. Details of the distance travelled, average speed and details of the veterinary inspection reported in the vet card at the time of completition (group P) or elimination (group NP).
