# Supplementary material for: Circulating Transcriptional Profile Modulation in Response to Metabolic Unbalance Due to Long-Term Exercise in Equine Athletes: A Pilot Study
Source: Genes (Basel). 2021 Dec 9;12(12):1965. doi: 10.3390/genes12121965 (PMC8701225; doi:10.3390/genes12121965)
Supplement: Supplementary file 1 [file genes-12-01965-s001.zip › supplementary/Table_S2.pdf]

genes

MDPI

Table S2. Hematology and Clinical Chemistry values differences between groups P and NP.

	Parameters	P Mean	NP Mean	Difference NP *vs* P	*p*-value
Hematology	WBCs (*10^3/μL)	12.9	8.6	-4.3	0.004
	RBCs (*10^6/μL)	9.6	12.0	2.4	1.53×10^{-4}
	Hb (g/dL)	15.1	24.3	9.2	5.83×10^{-7}
	Hct (%)	40.7	67.8	27.0	2.63×10^{-7}
	PLT (*10^3/μL)	157.9	195.7	37.8	0.144
	MCV (fl)	42.8	46.4	3.7	0.008
	MCH (pg)	15.9	15.3	-0.6	0.143
	MCHC (%)	37.2	32.9	-4.3	7.71×10^{-5}
	RDW (%)	23.3	19.5	-3.8	0.016
	MPV (fl)	6.1	6.0	0.0	0.479
	% Lymphocytes	15.1	11.5	-3.6	0.082
	% Monocytes	2.6	1.8	-0.7	0.101
	% Granulocytes	82.0	86.7	4.7	0.078
	# Lymphocytes	1.3	0.9	-0.4	0.013
	# Monocytes	0.1	0.1	0.0	0.238
	# Granulocytes	12.2	7.6	-4.6	0.024
Clinical Chemistry	Urea (mg/dl)	47.3	61.6	14.3	0.018
	Creatinine (mg/dL)	1.7	2.4	0.7	0.001
	Total bilirubin (mg/dL)	5.0	4.9	-0.1	0.938
	Direct bilirubin (mg/dL)	0.4	0.3	-0.1	0.603
	Indirect bilirubin (mg/dL)	4.5	4.5	0.0	0.985
	AST (IU/L)	451.4	576.4	125.0	0.085
	AST/km (IU/L)*	5.4	5.9	0.4	0.654
	GGT (IU/L)	15.0	15.6	0.6	0.804
	CK (IU/L)	2411.8	2823.5	411.8	0.699
	CK/km (IU/L)*	22.5	30.8	8.3	0.547
	LDH (IU/L)	1252.8	1498.3	245.5	0.264
	LDH/km (IU/L)*	14.9	15.8	0.9	0.817
	Total plasma proteins (g/dL)	7.4	8.5	1.1	3.82×10^{-4}
	Albumin (g/dL)	4.3	4.7	0.4	0.005

* These values were also normalized for the raced distance.
