# Supplementary material for: Circulating Transcriptional Profile Modulation in Response to Metabolic Unbalance Due to Long-Term Exercise in Equine Athletes: A Pilot Study
Source: Genes (Basel). 2021 Dec 9;12(12):1965. doi: 10.3390/genes12121965 (PMC8701225; doi:10.3390/genes12121965)
Supplement: Supplementary file 1 [file genes-12-01965-s001.zip › supplementary/Table_S3.pdf]

genes

MDPI

Table S3. Statistics of sequencing and mapping.

Sample ID	Reads after trimming	Uniquely Mapped on spike-in	%	Uniquely Mapped on miRNA (mirbase22)	%	Uniquely Mapped on genome (equcab3)	%
P1	15,698,407	9,444	0.06	1,373,474	8.75	2,363,204	16.55
P2	4,020,540	2,490	0.06	989,087	24.62	635,793	21.17
P3	10,420,107	3,228	0.03	5,122,653	49.18	550,574	12.11
P4	18,604,477	6,632	0.04	11,252,629	60.51	462,384	6.54
P5	19,195,414	5,148	0.03	1,476,715	7.70	1,383,126	7.82
P6	16,993,953	11,232	0.07	12,847,257	75.65	693,555	17.32
P7	6,866,968	5,746	0.08	5,708,927	83.21	164,614	14.75
P8	17,357,639	8,198	0.05	10,268,597	59.19	1,182,511	17.51
P9	10,890,730	21,764	0.2	7,028,060	64.66	915,909	25.82
P10	27,130,659	9,997	0.04	13,706,864	50.54	1,451,798	11.36
NP1	26,045,724	16,587	0.06	5,756,368	22.12	1,469,640	7.47
NP2	9,278,343	786	0.01	5,346,534	57.63	283,818	7.32
NP3	12,280,022	16,012	0.13	6,000,279	48.93	755,481	12.33
NP4	9,794,772	3,090	0.03	1,812,365	18.51	445,557	5.62
NP5	20,300,578	7,022	0.03	16,608,950	81.84	237,707	6.79
NP6	5,380,246	1,287	0.02	734,693	13.66	318,622	6.94
NP7	15,295,203	2,436	0.02	6,648,444	43.47	733,906	8.69
NP8	11,564,159	1,353	0.01	6,755,043	58.42	460,032	10.25
**Average**	**14,284,330.06**	**7,358.44**	**0.05**	**6,635,385.50**	**46.03**	**806,012.83**	**12.02**
